# “Cancer 2015”: A Prospective, Population-Based Cancer Cohort—Phase 1: Feasibility of Genomics-Guided Precision Medicine in the Clinic

**DOI:** 10.3390/jpm5040354

**Published:** 2015-10-29

**Authors:** John P. Parisot, Heather Thorne, Andrew Fellowes, Ken Doig, Mark Lucas, John J. McNeil, Brett Doble, Alexander Dobrovic, Thomas John, Paul A. James, Lara Lipton, David Ashley, Theresa Hayes, Paul McMurrick, Gary Richardson, Paula Lorgelly, Stephen B. Fox, David M. Thomas

**Affiliations:** 1 Division of Cancer Research, Peter MacCallum Cancer Centre, 7 St Andrews Place, East Melbourne VIC 3002, Australia; E-Mails: heather.thorne@petermac.org (H.T.); ken.doig@petermac.org (K.D.); stephen.fox@petermac.org (S.B.F.); d.thomas@garvan.org.au (D.M.T.); 2 Sir Peter MacCallum Department of Oncology, The University of Melbourne, Parkville VIC 3010, Australia; 3 Department of Pathology, Peter MacCallum Cancer Centre, East Melbourne VIC 3002, Australia; E-Mail: andrew.fellowes@petermac.org; 4 Department of Epidemiology and Preventative Medicine, Alfred Centre, Monash University, Prahran VIC 3181, Australia; E-Mails: mark.lucas@monash.edu (M.L.); john.mcneil@monash.edu (J.J.M.); 5 Centre for Health Economics, Monash University, Clayton VIC 3800, Australia; E-Mails: brett.doble@monash.edu (B.D.); paula.lorgelly@monash.edu (P.L.); 6 Translational Genomics and Epigenomics Laboratory, Olivia Newton-John Cancer Research Institute, Austin Health, Heidelberg VIC 3084, Australia; E-Mail: alex.dobrovic@onjcri.org.au; 7 Department of Pathology, The University of Melbourne, Parkville VIC 3010, Australia; 8 School of Cancer Medicine, La Trobe University, Bundoora VIC 3084, Australia; 9 Medical Oncology, Olivia Newton-John Cancer Research Institute, Austin Health, Heidelberg VIC 3084, Australia; E-Mail: Tom.John@onjcri.org.au; 10 Division of Cancer Medicine, Peter MacCallum Cancer Centre, East Melbourne VIC 3002, Australia; E-Mail: paul.james@petermac.org; 11 Department of Medical Oncology, The Royal Melbourne Hospital, Melbourne Health, Parkville VIC 3010, Australia; E-Mail: lara.lipton@mh.org.au; 12 The Andrew Love Cancer Centre, Geelong Hospital, Barwon Health, Geelong VIC 3220, Australia; E-Mail: david.ashley@BarwonHealth.org.au; 13 Warrnambool Hospital, SouthWest Healthcare, Warrnambool VIC 3280, Australia; E-Mail: thayes@westvic.com.au; 14 Department of Surgery, Cabrini Institute, Cabrini Health, Malvern VIC 3144, Australia; E-Mail: pjm@colorectal.com.au; 15 Haematology and Oncology, Cabrini Institute, Cabrini Health, Malvern VIC 3144, Australia; E-Mail: gary.richardson@ocv.net.au; 16 The Kinghorn Cancer Centre and Garvan Institute, Victoria Street, Darlinghurst 2010, NSW, Australia

**Keywords:** cancer genomics cohort, next-Gen sequencing, precision medicine, health economics

## Abstract

“Cancer 2015” is a longitudinal and prospective cohort. It is a phased study whose aim was to pilot recruiting 1000 patients during phase 1 to establish the feasibility of providing a population-based genomics cohort. Newly diagnosed adult patients with solid cancers, with residual tumour material for molecular genomics testing, were recruited into the cohort for the collection of a dataset containing clinical, molecular pathology, health resource use and outcomes data. 1685 patients have been recruited over almost 3 years from five hospitals. Thirty-two percent are aged between 61–70 years old, with a median age of 63 years. Diagnostic tumour samples were obtained for 90% of these patients for multiple parallel sequencing. Patients identified with somatic mutations of potentially “actionable” variants represented almost 10% of those tumours sequenced, while 42% of the cohort had no mutations identified. These genomic data were annotated with information such as cancer site, stage, morphology, treatment and patient outcomes and health resource use and cost. This cohort has delivered its main objective of establishing an upscalable genomics cohort within a clinical setting and in phase 2 aims to develop a protocol for how genomics testing can be used in real-time clinical decision-making, providing evidence on the value of precision medicine to clinical practice.

## 1. Introduction

Despite recent data showing trends of improving cancer survival rates worldwide, cancer is now the leading cause of disease burden and mortality. Indeed, incidence rates are increasing such that the number of new cases is projected to increase from 14.1 million in 2012 to almost 25 million over the next two decades [1]. This projected rise of ~75% in cancer cases coincides with the advent of “personalized, precision or stratified” medicine, with an increasing number of molecularly targeted therapies in development and receiving approval to treat tumours in patients with defined, “actionable” genetic mutations. This shift towards targeted therapies is not only challenging the delivery of oncology and pathology services but also wider aspects of the health care system, including the funding model, particularly as these therapies are expensive, which combined with a growing incidence of cancer is likely to contribute to escalating health costs that are unsustainable.

Despite a sense of inevitability, there remain a number of research and clinical questions that need to be addressed to appropriately prepare for the era of genomic medicine. With this in mind, “Cancer 2015” was devised to establish a prospective and longitudinal, population-based cancer genomic cohort with the main purpose of determining how molecular pathology could be incorporated into the routine care of cancer patients so that they could benefit from molecularly targeted therapies in place of often toxic and ineffective chemotherapy agents. The main objectives of Cancer 2015 are summarized as follows:

- Establishment of a database consisting of biospecimens as well as clinical and epidemiological data to be used as a clinical and research resource.
- To screen cancer tumour DNA isolated from diagnostic tissue samples from patients independent of cancer subtype, to determine if any mutations are present that may potentially be of clinical and therapeutic significance.
- To yield data on the total population frequency of these mutations and identify patients that may benefit from therapeutics targeted against these “actionable” mutations.
- To collect health-related quality of life (HRQoL) responses longitudinally, and link in administrative health care resource use and cost data, in order to facilitate the assessment of the value of targeted therapies, and cancer care more generally.

Phase 1 (pilot) of the study commenced in November 2011 with the overall objective to recruit a minimum of 1000 new incident cancers from five hospitals in the state of Victoria [2] (which comprises ~25% of the Australian population). All solid cancers independent of histotype, were included in order to reflect the cancer burden sampling from a cross-section of the community representing both metropolitan and regional patients. The pilot was successful in recruiting over 1600 patients to the end of September 2014, from both metropolitan and regional hospitals. Data presented herein demonstrate our progress to date, describing preliminary, proof-of-principle results of one of only a few population-based genomic cohorts that are being undertaken worldwide [3,4,5].

## 2. Results

### 2.1. Cohort Characteristics

**Figure 1:**
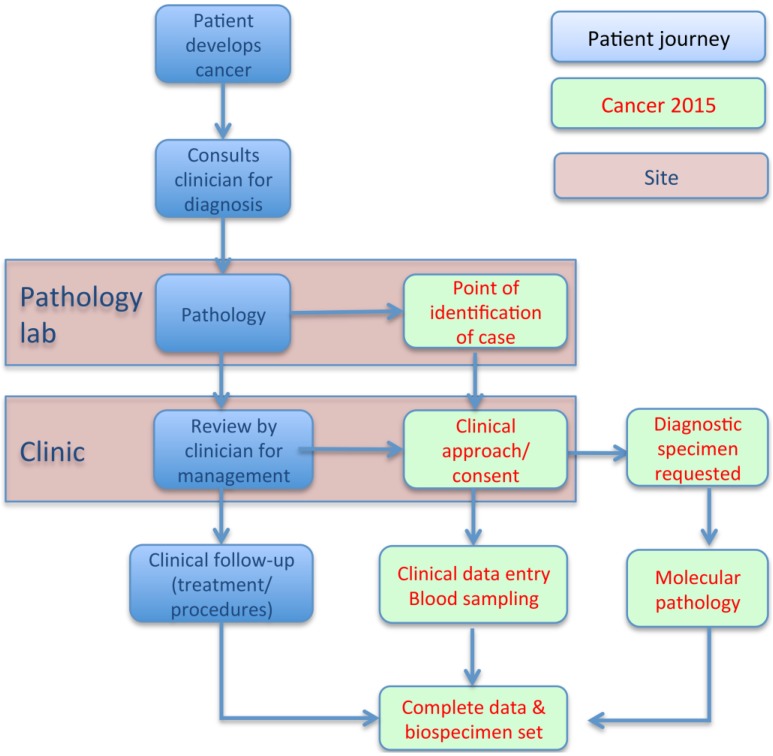
Schematic of pathway/protocol of patient recruitment to the cancer 2015 cohort.

The cohort recruited patients using a study protocol as summarized in Figure 1. Between November 2011 and September 2014 (phase 1), 1685 patients with newly diagnosed cancers were consented into the Cancer 2015 Cohort, surpassing its milestone target of 1000. The patient demographics and some of their clinical characteristics are shown in Table 1. Approximately 34% of participants were between 61–70 years of age at diagnosis with a median age of 63.2 (range 19–92) years, compared with the average age of cancer diagnosis in Australia reported as 65.4 years in 2009 [6]. Recruitment was generally balanced by gender, with the exception of the Peter MacCallum Cancer Centre, which is a specialist cancer treatment centre for many male-dominated cancer types as well as a tertiary radiotherapy centre. The median period of time in which patients were enrolled into the Cancer 2015 cohort after diagnosis was 26 days. Of the 1685 participants in the cohort at the end of phase 1, 233 were deceased (13.8%), whilst a further 63 (3.7%) had requested to withdraw from the study either in written or verbal form (Table 1).

**Table 1:** Demographic characteristics of cancer 2015 cohort participants.

Characteristic	N	%
*Total Consented:*	1685	
Deceased	233	13.8
Withdrawn	63	3.7
Male	916	54.0
Female	769	45.5
*Recruited from Institution:*		
Cabrini Hospital	322	19.0
Geelong Hospital	284	16.7
Peter MacCallum Cancer Centre	523	30.9
Royal Melbourne Hospital	362	21.5
Warrnambool Hospital	194	11.5
*Regional Statistics:*		
Metropolitan	936	57.7 (69)
Non-Metropolitan	685	42.3 (31)
*Age (years and 10-yr deciles)*		
Median	63.2	
11–20	1	0.1
21–30	27	1.6
31–40	73	4.4
41–50	204	12.2
51–60	374	22.3
61–70	573	34.2
71–80	317	18.9
81–90	103	6.1
91–100	3	0.2
*Region of Origin (Birth):*		
Africa	22	1.3 (2.5)
Asia	70	4.2 (4.8)
Australia (inc. Oceania)	1222	72.5 (67.6)
Europe	268	15.9 (24.3)
North America	9	0.5 (0.4)
South America	2	0.1 (0.4)
Marital Status*:		
Never married	69	4.1
Married	803	47.7
Divorced	97	5.8
Widowed	108	6.4
Separated	19	1.1
Not stated	575	34.1
Education Level*:		
Primary	51	3.0
Junior Secondary	168	10.0
Senior Secondary	148	8.8
Graduate	105	6.2
Post-graduate	44	2.6
No formal education	9	0.5
Not stated	1146	68.0
*Disease Presentation Mode:*		
Symptomatic	1081	64.2
Asymptomatic/incidental	196	11.6
Screening	361	21.4
Not Stated	38	2.3
*Performance Status (ECOG):*		
0	1066	63.3
1	400	23.7
2	121	7.2
3	42	2.5
4	4	0.2
*Charlson Co-Morbidities Index:*		
0–5	1448	85.9
6–10	58	3.4
>10	170	10.1
*Private Hospital Insurance:*		
Yes	694	41.2
No	948	56.3
*Smoking Status:*		
Daily	209	12.4
Weekly	13	0.8
Irregular	25	1.5
Ex-smoker	747	44.3
Never Smoked	630	37.4
*Past History of Cancer:*		
Yes	303	18.0
No	1336	79.3
*Family History of Cancer (1st/2nd order blood relative):*		
Yes	1106	65.6
No	507	30.1
Hereditary Syndromes	10	0.6
*Blood Samples Obtained:*		
Received	1505	89.3
Unavailable/Insufficient tissue	172	10.2
*Quality of Life Questionnaire responses:*		
Baseline	1606	95.3
Follow-up	1271	75.4
Medicare/Pharmaceutical Benefit Scheme Co-Consent	1590	94.4

*: Data capture of marital status and level of education was not initiated until 12 months after cohort start; Percentages in parentheses where given represent proportions of the population of Victoria [2].

**Figure 2:**
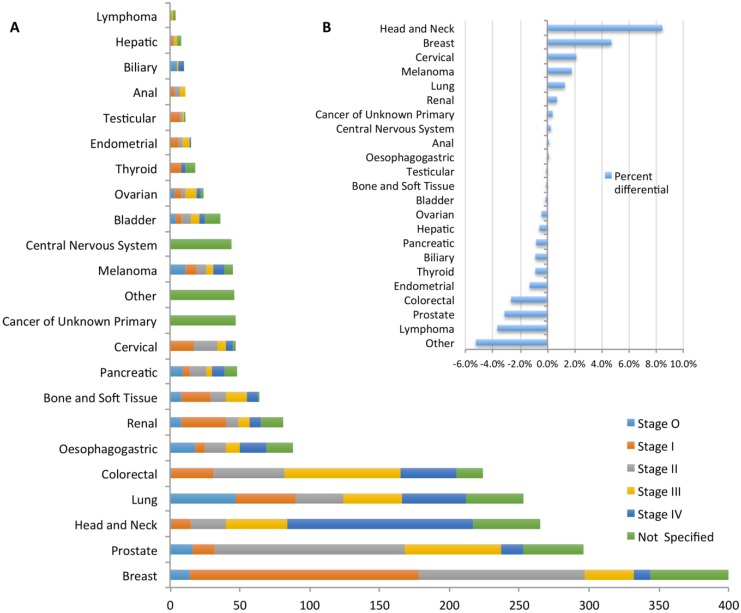
(**A**) Patient diagnoses in the Cancer 2015 Cohort separated into different tumour histo-types and clinical stage (as colour labelled in legend). Note: A minority of patients (n = 50) have tumours that fall into less common cancers such as skin, vulvovaginal, penile and urothelial cancers; (**B**) The variation between the Cancer 2015 Cohort accrual rate, expressed as a percentage of total cancers and separated by cancer histotype, compared with the VCR 2011 census of cancer incidence in Victoria (solid-cancers only; removal of paediatric and haematological cancers; Note: Melanoma incidences represent advanced stages only). Positive percentage differential reflect recruitment of patients with stated cancer histotype greater than published Victorian incidences.

Figure 2A presents the various solid cancer histotypes and associated clinical cancer stage of the patients in Phase 1. Whilst the cohort successfully accrued the expected major cancer histotypes such as breast, lung, colorectal and prostate cancer, it has also managed to recruit a substantial number of patients with less common cancers such as head and neck, bone/soft tissue (BST), renal, bladder and, to a lesser extent, cancer of unknown primary. A comparison of Cancer 2015 histotype numbers with the incidence in Victoria is presented inset as Figure 2B. These comparisons are depicted as the difference between the Cancer 2015 Cohort accrual rate and the Victorian Cancer Registry (VCR) 2011 census of cancer incidence [2]. Thus, zero percent differential depicts identical rates between the cohort and registry.

Whilst the cohort was mostly proportionate to the VCR census data of 2011, the rates of accrual of some types of cancer may have been biased by the cohort’s initial selection of hospitals. Notably there is a slight (<−1%) under-representation, in general, of gynaecological cancers due to the lack of a gynaecological cancer centre in Phase 1. Conversely, the cohort has a higher than expected rate of accrual of patients with head and neck cancers (>+8%) most probably due to the Peter MacCallum Cancer Centre being one of the major radiotherapy centres for Victoria. Expansion to a broader range of hospital oncology services should correct for any other ascertainment biases in future phases. The cohort currently offers a fairly even representation of each cancer-stage (I–IV; data not shown).

### 2.2. Data Completion

The clinical data elements collected by the Cancer 2015 Cohort were centred on a modified Core Clinical Cancer Data Set [7] as published by the Australian Institute of Health and Welfare (AIHW) and National Cancer Control Initiative 2004. At least 80% of these individual data elements have been fully completed after audit of the data (excluding optional fields) in the registry (Supplementary Table S1). Ongoing data auditing and cleansing will significantly improve these completion rates as the pilot matures as well as determine the accuracy of data collection via random sampling audit.

Cancer stage, as described above, was collected in >85% of the patient cohort, additional indicators such as the ECOG [8] performance status (95% complete) and Charlson Index [9] of co-morbidities (90% complete) yield data on the patient’s general capabilities and predictive mortality. Note that the majority of the cohort (62%) belong to ECOG category 0 (data not shown); described as “Fully active, able to carry on all pre-disease performance without restriction”, while 80% of patients have a Charlson Index score of 1 or less, indicative of a low level of co-morbid disease. These data will be important for identifying patients who may be suitable for clinical trials (see discussion).

### 2.3. Genomic Assay of Biospecimens

**Figure 3:**
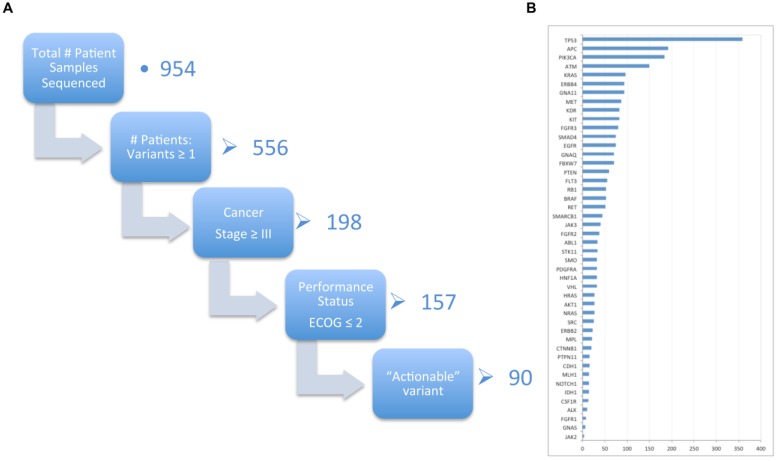
(**A**) The number of Cancer 2015 patients that have had tumour sample DNA sequenced, binned into those with at least one gene somatic variant identified; having advanced cancer (Stage Group ≥3); having an Eastern Co-operative Oncology Group (ECOG) Performance status of ≤2 (“no worse than ambulatory and capable of all self care but unable to carry out any work activities. Up and about more than 50% of waking hours”) and finally the variant(s) are deemed “actionable” (*i*.*e*., approved drugs available or drugs in clinical trials); (**B**) Comparison of the number of mutations observed per gene represented overall across all solid tumour histo-types in the cohort.

The majority of patients (94%) who have consented to Cancer 2015 had an initial blood sample drawn and processed into plasma and blood/buffy coat pellets as part of the recruitment protocol. Patient poor health was the main reason that samples were not obtained. Furthermore, approximately 10% of these patients with a base blood sample prior to treatment also have a repeat blood sample obtained at either the 3–6 or 12 month follow-up time points. With respect to tumour pathology samples, 1505 patient samples have been received from the 1685 patients recruited (90%) in phase 1. In a cross-section of 1157 samples received mid-phase 1 depicted in Figure 3A, 954 successfully yielded DNA of sufficient quantity and quality for the TruSeq™ Cancer gene variant testing (Illumina, San Diego, CA, USA), of which 556 samples (58%) had at least one somatic variant identified, noting that 398 had no somatic variant identified. A negative result using this assay may arise due to a disease causing somatic variant occurring outside the regions targeted by the assay. In addition the analytical process employed in this study was not suitable for detecting copy number variations or structural rearrangements. For the remaining samples (17.5%), testing was not possible due to quality control issues (typically DNA < 10 ng/µL) or lack of tissue availability. Furthermore, there were a number (90) of advanced cancer patients (Figure 3A) identified with gene variants deemed potentially “actionable”, defined as relatively healthy individuals by ECOG rating who may have been eligible for clinical trials of new targeted therapies or with previously approved drugs. Improvement to the pathology tissue requisition process is ongoing and involves either increasing the number of slides requested of tumour samples for some tumour types, limited macro-dissection of the tumour from “normal” tissue and/or quality assurance enhancements in the process or sensitivity of the variant assay testing in updated release versions (Illumina TruSight™) using optimised chemistry. This is discussed in detail in the publication centred on the molecular pathology/genomics aspects of the cohort [10], though an overall impression of the rates of gene mutations using our TruSeq™ cancer gene panel is depicted in Figure 3B where it is observed that not unexpectedly, TP53 is the most frequently mutated gene within our solid cancer sample collection from phase 1.

### 2.4. Patient Follow Up

**Figure 4:**
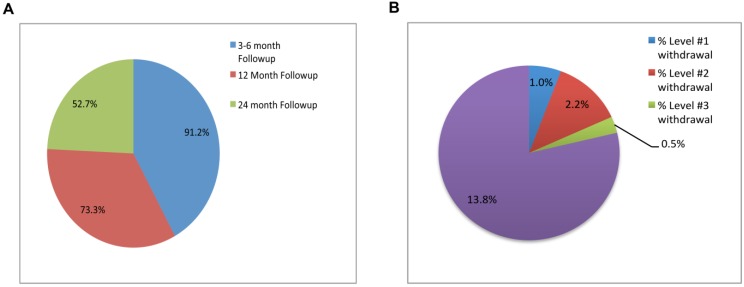
(**A**) The percentage of Cancer 2015 patients with at least one follow-up, binned into time periods 3–6, 12 and 24 months post-registration into phase 1 of the study; (**B**) Percentage of Cancer 2015 patients that have either deceased or withdrawn consent to the study as of end of phase 1 (level 1 = no contact but patient approves continuation of collecting data, level 2 = no contact but patient requests to stop collecting data, level 3 = no contact and patient requests removal of data and destruction of biospecimens).

The longitudinal nature of the cohort is an important asset of Cancer 2015. Follow-up with patients is progressing well such that the rate of successful follow-up is over 90% at 3–6 month post-consent and approximately 75% at 12 months (Figure 4A). Median first follow up interval is 5.9 months, with an overall median of 8.7 months (includes multiple follow-ups; data not shown). An analysis of overall survival rates for patients in the Cancer 2015 cohort at time points of 12, 24 and ~36 months post study start-up, finds 92.9%, 90.5% and 86.2% of patients alive, respectively, with a small number of patients having requested to be withdrawn from the study (Figure 4B), the majority preferring no further contact with the study (but satisfied for research to continue with their samples) for reasons such as their cancer is in remission and thus a desire to move on. The cohort has achieved a high compliance rate with respect to follow up PROM questionnaires (~75% at first follow-up; data not shown) using return-paid postal correspondence. These repeat time points for the HRQoL questionnaires will be pivotal for determining health outcomes over time, including quality adjusted life years (QALYs) which are often used in health economic evaluations. These outcomes, together with the resource use and costs derived from the linkages with the Medicare Benefits Schedule (MBS) and the Pharmaceutical Benefits Scheme (PBS); and hospitalisation records, offer the necessary evidence to begin to understand the economic implications of targeted cancer therapies using genomic testing.

Dependent on future funding, Cancer 2015 plans to increase its rate of biospecimen follow-up to form a resource pivotal for emerging areas such as circulating tumour DNA plasma biomarker studies [11]. To date, additional follow-up blood samples have been obtained from 152 individuals.

## 3. Discussion

In Phase 1 of this study, Cancer 2015 was set the task of establishing the feasibility of its program of work. It has successfully implemented a prospective, cancer patient recruitment program across five hospitals within the state of Victoria (Australia). The feasibility of patient accrual across hospitals from wide geographic distances encompassing both metropolitan (Melbourne and Geelong) and regional cities (Warrnambool) has been successfully demonstrated. This is important to prove ease of access to genomic testing for patients recruited from large and diverse geographic regions. The molecular pathology component of the first phase of Cancer 2015 demonstrated the feasibility of adopting next generation sequencing for mutational profiling of tumour samples [12] from Victorian metropolitan and regional centres and importantly delivered on its two main outcomes of test numbers and the viability of cancer gene panel testing. The longitudinal follow up of clinical data, HRQoL responses, together with data linkage of health care resource use has also been achieved, and it will ultimately yield evidence to evaluate the cost, outcomes and value of integrating genomics-guided interventions into standard care and treatment.

Notably, Cancer 2015 has provided data to support genomics-targeted recruitment of patients into clinical trials. Near the midpoint of phase 1, 90 advanced stage cancer patients have been identified as having “actionable” somatic gene variants that potentially could be treated either via approved drugs or those currently in clinical trials. Furthermore, initial analyses of the cohort data have demonstrated significant differences in mutation type and frequency compared with those previously reported by institutional series [12].

Apart from patient accrual in the major cancer histotypes, the cohort has also achieved significant accrual of patients with rarer and more importantly, less well-studied (and understood) cancers such as head and neck (H&N), bone and soft tissue (BST) and cancer of unknown primary (CUP). As such the cohort is well placed to provide unique data to support a new classification for at least some cancers (e.g., H&N, CUP), and this may open unexpected avenues for investigation and treatment in these poor prognosis tumours. The cohort has also identified actionable mutations in tumours not usually thought to harbour such genetic changes, raising new therapeutic opportunities, e.g., Ras-Raf or Akt-PI3K pathway in CUP. The breadth and depth of the cohort, in terms of coverage of cancer and the identification of new mutations will provide a platform from which to undertake unique health economic analyses of cancer care and more specifically targeted therapies.

A key goal of the current, Phase 2 of the Cancer 2015 cohort is to implement genomic testing/screening on a population-level for Victorian cancer patients in real-time (turn around time < 10 working days) for a wide range of clinically actionable somatic mutations using next generation sequencing. Advances in genome technology continue apace. Thus a significant challenge facing Cancer 2015 is to ensure a clinically robust and valid assay can continue to be delivered while incorporating relevant advances in cancer genomics and analytical genomic technology. The identification of actionable mutations will aid clinical cancer care by identifying approved, targeted cancer therapeutics and for many patients it will increase access to revolutionary clinical trials currently underway worldwide [13,14,15,16,17,18].

## 4. Experimental Section

Given the complex nature of establishing a multi-site, multi-histotype cohort, Cancer 2015 was first funded (by the Victorian Cancer Agency) as a pilot study. The feasibility and basic infrastructure was established in Phase 1 to enable patient recruitment to commence at five Victorian hospital sites; two capital city public hospitals, Peter MacCallum Cancer Centre and The Royal Melbourne Hospital; a city private hospital, Cabrini Hospital; and non-capital metropolitan and regional public hospitals, Geelong (Barwon Health) and Warrnambool Hospitals (SouthWest Health), respectively.

### 4.1. Study Design

Figure 1 provides an outline of the protocol for prospective patient recruitment into this study. Once a patient was diagnosed with cancer after pathology assessment and subsequent referral for review to a clinician or surgeon, those that appropriately conformed to the Cancer 2015 eligibility criteria (see below) after review of medical records, were approached for their consent to this study by interview. Each hospital participating in the Cancer 2015 Cohort was resourced by 1–2 research nurses, with the responsibility for co-ordinating and performing patient consents onsite and the centralization of the cohort administration and database within the study centre (Peter MacCallum Cancer Centre). Eligible patients included those with a new, pathologically confirmed diagnosis of solid-tumour cancer, independent of cancer histotype and stage; tumour accessible to biopsy and ideally prior to interventions such as surgery, chemo- and/or radiotherapy having commenced; and with a previous history of cancer being permitted. Patients were excluded if they were under 18 years of age or otherwise incapable of informed consent; also if the diagnosed cancer was most likely a recent (<12 months) recurrence of a previous incidence. This study was conducted in accordance with the Declaration of Helsinki and approved by the Human Research Ethics Committees of all the participating hospitals.

### 4.2. Biospecimens

Eligible patients who consented agreed to the collection of their diagnostic archival tumour block from pathology laboratories for the purposes of next-generation sequencing of the DNA isolated from the tumour tissue for targeted cancer gene panels. Moreover, patients also consented to the collection of 18 mL of blood for the purposes of distinguishing somatic from germline variants, if identified. However, apart from validation assays germline variants were not routinely tested during phase 1 due to prohibitive assay cost. Blood samples were processed as soon as possible (b/w 2–4h at Peter MacCallum and Royal Melbourne Hospital but often the next day from other participating hospitals), fractionating into either plasma and blood cell pellets or plasma and buffy coat aliquots. The potential benefits and implications associated with the use of tumour and/or blood DNA for the patient and their immediate family were clearly articulated both in the patient information and consent form and by the research nurse involved in their recruitment into the cohort. Patients have the capacity to refuse to be contacted if research results are identified that has implications for them or their families according to the principles within the National Statement on Ethical Conduct in Human Research [19].

During the pilot phase of the study, the Molecular Pathology Department at Peter MacCallum Cancer Centre performed mutational analysis on genomic DNA extracted from tumours using the Illumina TruSeq™Amplicon Cancer Panel (TSACP) next-generation targeted exome screen using ~150 ng DNA per sample on the MiSeq platform [12]. This technology choice arose from the comprehensive hot-spot focus, FFPE compatibility, and low cost afforded by this product. Although Illumina’s TSCAP lacks sensitivity to copy number and structural changes, these activating events can be assayed cost-effectively using alternative testing modalities. Specific mutations (212 in total across 48 genes) were screened using DNA extracted from formalin fixed, paraffin-embedded (FFPE) unstained sections. Analyses of the technical considerations of identifying mutations screened have previously been published [10,12]. Assays were batched during these initial phase 1 collections where the emphasis was on feasibility. As such, the results of genomic cancer gene panel tests where not returned to clinicians in a timely fashion to guide patient treatment (except in rare instances where clinicians specifically requested fast-tracking of results) and any somatic variants identified were returned as a research finding.

### 4.3. Data Collection

Patients’ medical records were reviewed such that the Cancer 2015 registry contained a minimum set of clinical data including tumour histo-type, site, morphology, laterality, stage, treatment summary (surgery, chemo-/radio-therapy) and intent (curative/palliative). In addition to patient demographics, the mode of presentation (symptomatic, incidental or screening), Charlson Index [19] of Co-morbidities from patient history, the Eastern Co-operative Oncology Group (ECOG) performance status [8] (clinically reported where possible) and a past history of cancer at both the personal and family (restricted to 2nd degree relatives) level, were all collected. Patients were asked to complete two patient reported outcome measures (PROMs) at the initial interview, the European Organization for Research and Treatment of Cancer (EORTC) QLQ-C30 [20] questionnaire and the EuroQol Group’s generic, preference based EQ-5D-3L [21] questionnaire. Collectively, these patient data, together with biospecimen sample identification, were housed in a secure, centralised database that was web-accessible across participating hospitals.

### 4.4. Data Linkages

Patients were also asked to consent to the extraction and linkage of individual data collated by the federal Department of Human Services (Australia) containing medical services and pharmaceutical resource use and expenditure (known respectively as the MBS and the PBS), as well as hospital administrative datasets provided by the Victorian Department of Health’s Data Linkages team. Such data of hospital admissions and emergency presentations of patients were provided to Cancer 2015 under several strict conditions (e.g., patient de-identification) regarding use and custodianship.

### 4.5. Patient Follow up

Patient follow up was performed at 6 and 12 months post-consent into the cohort, and continued every 12 months thereafter. This timeline was accelerated for a subset of the cohort (~10%) consisting of advanced cancer patients with an extra follow up added at 3 months post-consent. Further follow up will be performed over a period of 5 years (funding permitted) for all participants to monitor for response to treatments, progression/relapse and survival outcomes. Follow up involves gathering up-to-date clinical data from hospital and pathological records, and further PROM responses (majority via mail out of the set of questionnaires) to track health outcomes over time. Where possible, blood collection was repeated for a subset of patients who re-visited the participating hospitals near the follow up time points (within 1 month generally) for potential use in other studies which utilise plasma biomarkers for disease progression (e.g., circulating plasma tumour DNA dependent on protocol requirements).

## 5. Conclusions

It is increasingly clear that the implementation of a program of genomic medicine will require a population-level approach to screening and the identification of suitable cases for the future of clinical trials of targeted therapies [13,14]. Cancer 2015 has placed Victoria in an enviable position. It has a highly annotated clinical genomics registry whose data can be made available to ethically approved research upon application to the Steering Committee.

## Supplementary Materials

Please see Table S1: Cancer 2015 Registry Completeness by Data Element (as at 1 July 2015), Table S2: Cancer 2015_Supplementary List of Data Elements, Files: Cancer2015_Illumina_TSCA_Cancer Gene Panel Targets.xls, Cancer2015_Actionable Mutations_Current Targeted Drugs.xls.
