# Supplementary material for: “Cancer 2015”: A Prospective, Population-Based Cancer Cohort—Phase 1: Feasibility of Genomics-Guided Precision Medicine in the Clinic
Source: J Pers Med. 2015 Oct 29;5(4):354–69. doi: 10.3390/jpm5040354 (PMC4695860; doi:10.3390/jpm5040354)
Supplement: Supplementary file 1 [file jpm-05-00354-s001.zip › jpm-93434-supp-final.pdf]

Supplementary Materials

Table S1. Cancer 2015 registry completeness by data element as at 1 July 2015.

Data Element Collected	Percentage Complete (%)
PatientSecondaryContact_PostCode Complete	100.0
PatientPastHistoryOfCancer_CancerType Complete	100.0
PatientAddress_StreetNo Complete	100.0
PatientAddress_State Complete	100.0
PatientAddress_PostCode Complete	100.0
Patient_Surname Complete	100.0
Patient_PatientDOB Complete	100.0
Patient_GenderId Complete	100.0
Patient_FirstName Complete	100.0
Patient_DateOfBirth Complete	100.0
Patient_CountryOfBirth Complete	99.9
Patient_PatientURN Complete	99.6
PatientAddress_Suburb Complete	99.3
Patient_MedicareNumber Complete	99.1
PatientAddress_StreetName Complete	98.7
PatientPastHistoryOfCancer_TreatingInstitution Complete	98.7
PatientAddress_StreetNo Complete	98.6
Patient_IndigenousStatusId Complete	98.3
PatientAddress_TelephoneNumber Complete	97.6
PatientPresentation_HospitalInsurance Complete	97.0
PatientPresentation_PresentationMode Complete	96.6
PatientPresentation_PriorChemotherapy Complete	96.5
PatientPresentation_InfieldRadiotherapy Complete	96.5
PatientSecondaryContact_FirstName Complete	96.4
PatientPresentation_SmokingStatus Complete	95.9
PatientPresentation_ECOG Complete	95.5
PatientSecondaryContact_Surname	95.1
Patient_NextFollowupDate Complete	94.7
PatientSecondaryContact_TelephoneNumber Complete	92.5
Patient_ConsentForOtherStudies Complete	91.4
PatientPresentation_CharlsonIndex Complete	90.0
PatientSecondaryContact_Suburb Complete	80.7
PatientSecondaryContact_StreetName Complete	80.4
PatientSecondaryContact_StreetNo Complete	79.5
Patient_MaritalStatus Complete	73.1
PatientSecondaryContact_RelationshipToPatient Complete	71.3
PatientFamilyHistoryOfCancer_Relation Complete	68.4
Patient_OtherLanguage Complete	62.7
Patient_EducationAttained Complete	60.0
PatientAddress_EmailAddress Complete	33.5
PatientSyndrome_Syndrome Complete	2.3
DiagnosisTreatment_DiagnosisDate Complete	99.3
DiagnosisTreatment_TumourStream Complete	99.3
DiagnosisTreatment_TreatmentIntent Complete	98.5

Table S1. *Cont.*

Data Element Collected	Percentage Complete (%)
DiagnosisTreatment_TumourSite Complete	98.2
DiagnosisTreatment_ReferraltoPalliativeCareUnit Complete	98.1
DiagnosisTreatment_Radiotherapy Complete	97.4
DiagnosisTreatment_Chemotherapy Complete	97.2
DiagnosisTreatment_Morphology Complete	96.1
DiagnosisTreatment_FormalStage Complete	95.4
DiagnosisTreatment_CancerClinicalTrial Complete	94.9
DiagnosisTreatment_Surgery Complete	92.5
DiagnosisTreatment_AttachedReports Complete	90.7
DiagnosisTreatment_StagingSystem Complete	87.7
DiagnosisTreatment_Stage Complete	83.8
DiagnosisTreatment_StagingMethod Complete	68.8
FollowupOutcomes_Status Complete	100.0
FollowupOutcomes_FollowUpDate Complete	100.0
FollowupOutcomes_DiseaseRecurredOrProgressed Complete	99.9
FollowupOutcomes_NextFollowupDate Complete	97.9
FollowupOutcomes_ECOG Complete	97.1
FollowupOutcomes_TreatmentSummary Complete	91.8
FollowupOutcomes_Chemotherapy Complete	91.2
FollowupOutcomes_PallativeRefDate Complete	90.8
FollowupOutcomes_ReferraltoPalliativeCareUnit Complete	90.0
FollowupOutcomes_Radiotherapy Complete	88.8
FollowupOutcomes_CancerClinicalTrial Complete	81.7
FollowupOutcomes_Surgery Complete	81.4
FollowupOutcomes_CauseofDeath Complete	71.9
FollowupOutcomes_DiseaseRecurredOrProgressedDate Complete	29.2
FollowupOutcomes_DeathDate Complete	14.5
QualityofLifeSurveys_EQ-5DSurveyDate Complete	98.1
QualityofLifeSurveys_EQ-C30SurveyDate Complete	98.1
QualityofLifeSurveys_EQ-8DSurveyDate Complete	98.0
Biospecimens_BloodSampleNumber Complete	86.5
Biospecimens_BloodCollectionDate Complete	86.4
Biospecimens_SampleNumber Complete	78.1
Biospecimens_TumourCollectionDate Complete	77.4

Cancer 2015_List of Data Elements

1. Patient Registration

Table S2. Data elements and attributes.

**Element Name**	**Type** (Fixed/Text)	**Length**	**Format**	**Source** Self-reported=Y or Records=N
**Patient Details**				
ID Number	Text	12	nn.nn.nnnnn	Auto-generated
Surname	Text	250		Y
First Name	Text	250		Y
Middle Name	Text	20		Y
Date of birth	Date	Date picker	dd/mm/yyyy	Y
Gender	Single select Drop down	250		Y
Medicare Card Number	Number	10	nnnn-nnnnn-n	N
Country of birth	Single select Drop down	250		Y
Languages other than English	Text	250		Y
Indigenous Status	Single select Drop down			Y
Registered at Institution	Single select drop down			N
Email Address	Text	250		Y
Telephone Number	Number	10		Y
Unit No.	Text	20		Y
Street No.	Text	20		Y
Street Name	Text	250		Y
Suburb/Locality	Text	250		Y
State	Drop down			Y
Postcode	Number		nnnn	Y
**Secondary Contact**	Name and Address/email	As above	As above	Y
Consent for other studies	Drop Down	Y/N-Cancer or Other trial		Y
**Presentation**				
Presentation Mode	Single select Drop down			N
ECOG	Single select drop down			Y
Charlson Index	Number			Y

Table S2. *Cont.*

**Element Name**	**Type** (Fixed/Text)	**Length**	**Format**	**Source** Self-reported=Y or Records=N
Private Insurance	Single select Drop down (Y/N)			Y
Smoking Status	Single select Drop down			Y
Prior Chemotherapy	Single select Drop down (Y/N)			Y
Prior Radiotherapy	Single select Drop down (Y/N)			Y
Infield Radiotherapy	Single select Drop down (Y/N)			N
Past History of Cancer	Single select Drop down (Y/N)			Y
Cancer Type	Single select drop down			Y
Treating Institution	Text	250		Y
Age at diagnosis	Number		nn	Y
**Family History**				
Number of siblings	Number		nn	Y
Number of children	Number		nn	Y
Family History of cancer	Single select Drop down	(Y/N)		Y
Syndromes	Single select drop down	250		No
Cancer type	Single select drop down	250		No
Blood Relative (1st or 2nd degree)	Single select Drop down	250		Y
Age of Onset	Number			No

2. Diagnosis and Treatment

Table S2. *Cont.*

**Element Name**	**Type**	**Length**	**Format**	**Source**
	(Fixed/Text)			Self-reported=Y or Records=N
**Diagnosis**				
Date of Diagnosis	Date	Date picker	dd/mm/yyyy	N
Formal stage	Single select Drop down	250		N
Staging methods	Single select Drop down	250		N
Tumour Stream	Single select Drop down	250		N
Other (Stream)	Text			N

Table S2. *Cont.*

**Element Name**	**Type**	**Length**	**Format**	**Source**
	(Fixed/Text)			Self-reported=Y or Records=N
Tumour Site	Single select Drop down	250	ICD10AM	N
Laterality	Single select Drop down	250		N
Staging system	Single select drop down	250		N
Stage	Single select Drop down	250		N
Morphology	Single select Drop down	250	ICD10AM	N
Attached reports	Browse-add pdf	N/A	Pdf (2MB limit)	N/A
**Treatment Summary**				
Treatment Intent	Single select Drop down			N
Cancer Clinical Trial	Single select Drop down			N
Surgery	Single select Drop down	(Y/N)		N
Date	Date picker	dd/mm/yyyy		N
Primary Procedure	Single select Drop down		ICD10AM	N
Institution	Text	250		N
**Radiotherapy**	Single select Drop down	(Y/N)		N
Start Date	Date picker		dd/mm/yyyy	N
Target Site	Single select Drop down			No
Dose	Number	(Gy)	nn.nn	No
Institution	Text	250		N
Unit Record Number	Text	15		N
Timing	Single select Drop down	Adj, NeoAdj, Adv		N
Procedure	Single select Drop down	Brachy, 131I-Ablation,177 LuOct, SABR		
**Chemotherapy**	Single select Drop down (Y/N)	(Y/N)		N
Institution	Text	250		N
Start Date	Date picker		dd/mm/yyyy	N
Unit Record Number	Text	15		N
Timing	Single select Drop down	Adj, NeoAdj, Adv		N
Line	Number	250	nn…	
Regimen	Single select Drop down	250	Self-curated	N
Drugs	Single select Drop down	250	Self-curated	N
Referral to Palliative Care unit	Single select Drop down (Y/N)	(Y/N)		N
Start Date	Date picker		dd/mm/yyyy	N

3. FollowUP and OutComes

Table S2. *Cont.*

**Element Name**	**Type** (Fixed/Text)	**Length**	**Format**	**Source** Self-reported=Y or Records=N
**Patient Status**				
Follow-up date	Date picker		dd/mm/yyyy	Yes
Follow-up Number	Single select Drop down	Follow-up#	#1,2,3,4	
Status	Single select dropdown	250	Alive, Declined, Too unwell, Benign, Deceased, No Response Unknown	Yes
Date of death	Date picker		dd/mm/yyyy	No
Death Date Approximate	Tick-box	1	Ticked/or unticked	
Cause of death	Single select dropdown	250	Disease, Treatment, Other	No
ECOG	Single select dropdown	25	0-normal, 1-limited, 2-selfcare, 3-50%disabled, 4-100%disabled, 5-Death, **N/A–info N/A	No
**Disease Status**				
Disease recurred/progressed	Single select dropdown	(Y/N)	If Y, then fields below Treatment Summary open	No
Date detected	Date Picker		dd/mm/yyyy	No
Treatment summary	Single select Drop down		No Treatment, Ongoing 1st line, New treatment	
Cancer Clinical Trial	Single select Drop down		No trial Phase 1, Phase 2, Phase 3, Other Intervention, Epidemiol/registry, Psychosocial/sup, Other	N
Surgery	Single select Drop down	(Y/N)		N
Date	Date picker	dd/mm/yyyy		N
Primary Procedure	Single select Drop down		ICD10AM	N
Institution	Text	250		N
**Radiotherapy**	Single select Drop down	(Y/N)		N
Start Date	Date picker		dd/mm/yyyy	N
Target Site	Single select Drop down			No
Dose	Number	(Gy)	nn.nn	No
Institution	Text	250		N

Table S2. *Cont.*

**Element Name**	**Type** (Fixed/Text)	**Length**	**Format**	**Source** Self-reported=Y or Records=N
Unit Record Number	Text	15		N
Timing	Single select Drop down	Adj, NeoAdj, Adv		N
Procedure	Single select Drop down	Brachy, 131I-Ablation,177LuOct, SABR		
**Chemotherapy**	Single select Drop down (Y/N)	(Y/N)		N
Institution	Text	250		N
Start Date	Date picker		dd/mm/yyyy	N
Unit Record Number	Text	15		N
Timing	Single select Drop down	Adj, NeoAdj, Adv		N
Line	Number	250	nn…	
Regimen	Single select Drop down	250	Self-curated	N
Drugs	Single select Drop down	250	Self-curated	N
**Referral to Palliative Care unit**	Single select Drop down (Y/N)	(Y/N)		N
Start Date	Date picker		dd/mm/yyyy	N
Chemo drugs	Single select dropdown	250		No

4. Biospecimen and Mutation Results

Table S2. *Cont.*

**Element Name**	**Type** (Fixed/Text)	**Length**	**Format**	**Source** Self-reported=Y or Records=N
**Tumour Biospecimen**				
Identifier	Text box	250		Y
Collection Date	Date Picker		dd/mm/yyyy	Y
Tumour sample URN	Text box	250		Y
Number of Samples	Number	250	nnn…	Y
Source	Single select dropdown	25	Biopsy Resection	N
Mutation Tested	Single select dropdown	(Y/N)		Y

Table S2. *Cont.*

**Element Name**	**Type** (Fixed/Text)	**Length**	**Format**	**Source** Self-reported=Y or Records=N
Recorded Date	Date Picker		dd/mm/yyyy	Y
Mutation Result	Upload file		Csv and pdf	Y
**Blood Biospecimen**				
Identifier	Text box	250		Y
Collection Date	Date Picker		dd/mm/yyyy	Y
Other Identifier	Text box	250		Y
Number of Samples	Number	250	nnn…	Y
Source	Single select dropdown	25	Plasma Pellet	N
Mutation Tested	Single select dropdown	(Y/N)		Y
Recorded Date	Date Picker		dd/mm/yyyy	Y
Mutation Result	Upload file		Csv and pdf	Y

5. Quality Of Life Surveys

Table S2. *Cont.*

**QoL Surveys**				
**Element Name**	**Type** (Fixed/Text)	**Length**	**Format**	**Source** Self-reported=Y or Records=N
Period	Single select dropdown	25	Diagnosis, Follow Up1, “”2...	Y
QLQ-name	Tick-box		-C30, -8D, -5D	Y
Reason (missing)	Single select dropdown	25	N/A, No response	Y
Add Survey	Click to add		QoL responses	Y
Survey ID	Text	10	Auto-generated	N/A
Survey Date	Date picker		dd/mm/yyyy	Y
QoL Link	Single select dropdown	25	Link to “Period” above	Y
Missing Reason	Text	50	N/A No response	Y
Notes	Text	250	Free txt	Y
Date Created	Date Stamp		dd/mm/yyyy	N/A
Date Modified (last)	Date Stamp		dd/mm/yyyy	N/A

© 2015 by the authors; licensee MDPI, Basel, Switzerland. This article is an open access article distributed under the terms and conditions of the Creative Commons Attribution license (http://creativecommons.org/licenses/by/4.0/).
